# Beyond Colonization: An Atypical Presentation of Maternal Group B Streptococcus Infection

**DOI:** 10.7759/cureus.85225

**Published:** 2025-06-02

**Authors:** Sahith Kaki, Madison Natemeier, Michael Clark, Maciek Jurecki

**Affiliations:** 1 School of Osteopathic Medicine, Lake Erie College of Osteopathic Medicine, Erie, USA; 2 Family Medicine, Allegheny Health Network (AHN) St. Vincent Hospital, Erie, USA

**Keywords:** bacteremia, gbs infection, gbs prophylaxis, pregnancy, strep cellulitis

## Abstract

Group B Streptococcus (GBS), a commensal species that colonizes the genitourinary and GI tracts of many women, is well-studied as a leading cause of neonatal infection in the United States. Maternal colonization is the primary risk factor for neonatal sepsis, with vertical transmission from mother to fetus occurring during delivery. To reduce this risk, universal screening and intrapartum antibiotic prophylaxis have been implemented. While many women who test positive for colonization are asymptomatic, a small subset may develop invasive GBS infections such as bacteremia, meningitis, or pneumonia. In this report, we describe a rare presentation of invasive maternal GBS in a 32-year-old woman with GBS bacteremia and associated cellulitis. The patient was appropriately treated with antibiotics at the time of presentation and again during delivery. This case highlights the importance of recognizing and reporting atypical manifestations of GBS infection in pregnancy to better characterize the full spectrum of the disease.

## Introduction

Group B Streptococcus (GBS), or *Streptococcus agalactiae*, is a gram-positive bacterium that colonizes the genitourinary and GI tracts of 10-30% of women [1]. GBS is a leading cause of neonatal infection in the United States, responsible for approximately 30% of early-onset neonatal sepsis cases [2]. Maternal colonization is the primary risk factor for neonatal infection, acquired from vertical transmission during parturition [1]. Approximately 50% of colonizers will transmit the bacteria to their newborns, and 1-2% of those will develop GBS early-onset disease if not given intrapartum antibiotic prophylaxis (IAP) [1]. The incidence of neonatal GBS disease has decreased in recent decades, largely in part to routine screening and IAP [2]. Currently, the American College of Obstetricians and Gynecologists (ACOG) recommends universal screening with rectovaginal cultures between 36 0/7 and 37 6/7 weeks, with IAP indicated for patients who test positive [1].

Most colonized women remain asymptomatic; however, a minority develop invasive GBS disease, which the CDC defines as GBS found in sterile sites such as blood, CSF, and synovial fluid [3,4]. Some of the most common clinical manifestations of invasive GBS infections in adults include skin and soft tissue infections, bacteremia, pneumonia, urosepsis, and osteomyelitis [5]. Less commonly, meningitis and endocarditis have been reported [5]. Invasive GBS disease in pregnancy is uncommon, occurring in approximately 0.1% of all pregnancies [4]. While rare, these infections can be life-threatening, necessitating early recognition of infection and prompt intervention to avoid complications [5].

This case reports a presentation of invasive maternal GBS infection, evidenced by culture-confirmed bacteremia with associated cellulitis, in a patient who tested positive for rectovaginal GBS colonization. It contributes to the limited literature regarding invasive GBS in pregnancy, specifically cellulitis and bacteremia. Although cases of GBS colonization manifesting as skin and soft tissue infections are rare, their severity warrants greater clinical awareness and consideration in the differential diagnosis. These cases remain underreported, highlighting the need for continued documentation and further investigation into the full spectrum of invasive maternal GBS disease.

## Case presentation

**Figure 1:**
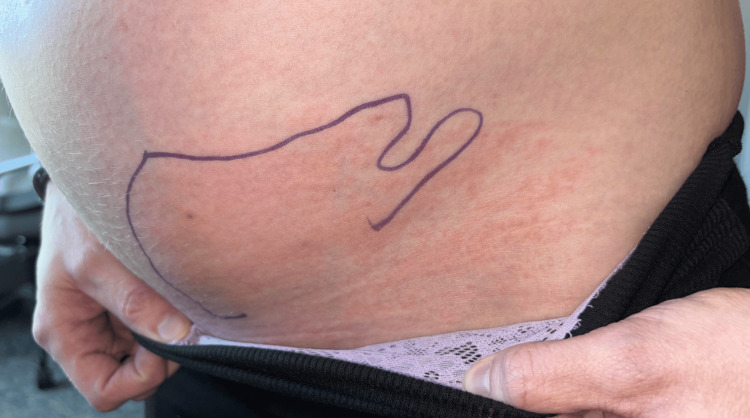
Photograph of the rash taken approximately five hours prior to presentation The borders were marked by the patient; note the extension beyond the superior and lateral margins.

A 32-year-old G2P1001 female at 32 weeks and one day gestational age presented to the labor and delivery triage due to a one-day history of a progressively enlarging erythematous, pruritic, and warm rash of the left lower abdominal quadrant (Figure 1), along with acute-on-chronic left labial edema resulting in pain with ambulation. Associated symptoms included headache, chills, and myalgias. Her past medical history was significant for asthma, primary lymphedema involving the left labia and all four extremities, lower extremity varicosities status post bilateral great saphenous vein ablation in 2015, and right nephrectomy in 2013 due to transplant donor status. Her previous pregnancy consisted of an uncomplicated course and GBS-negative status, resulting in a spontaneous vaginal delivery at term.

**Figure 2:**
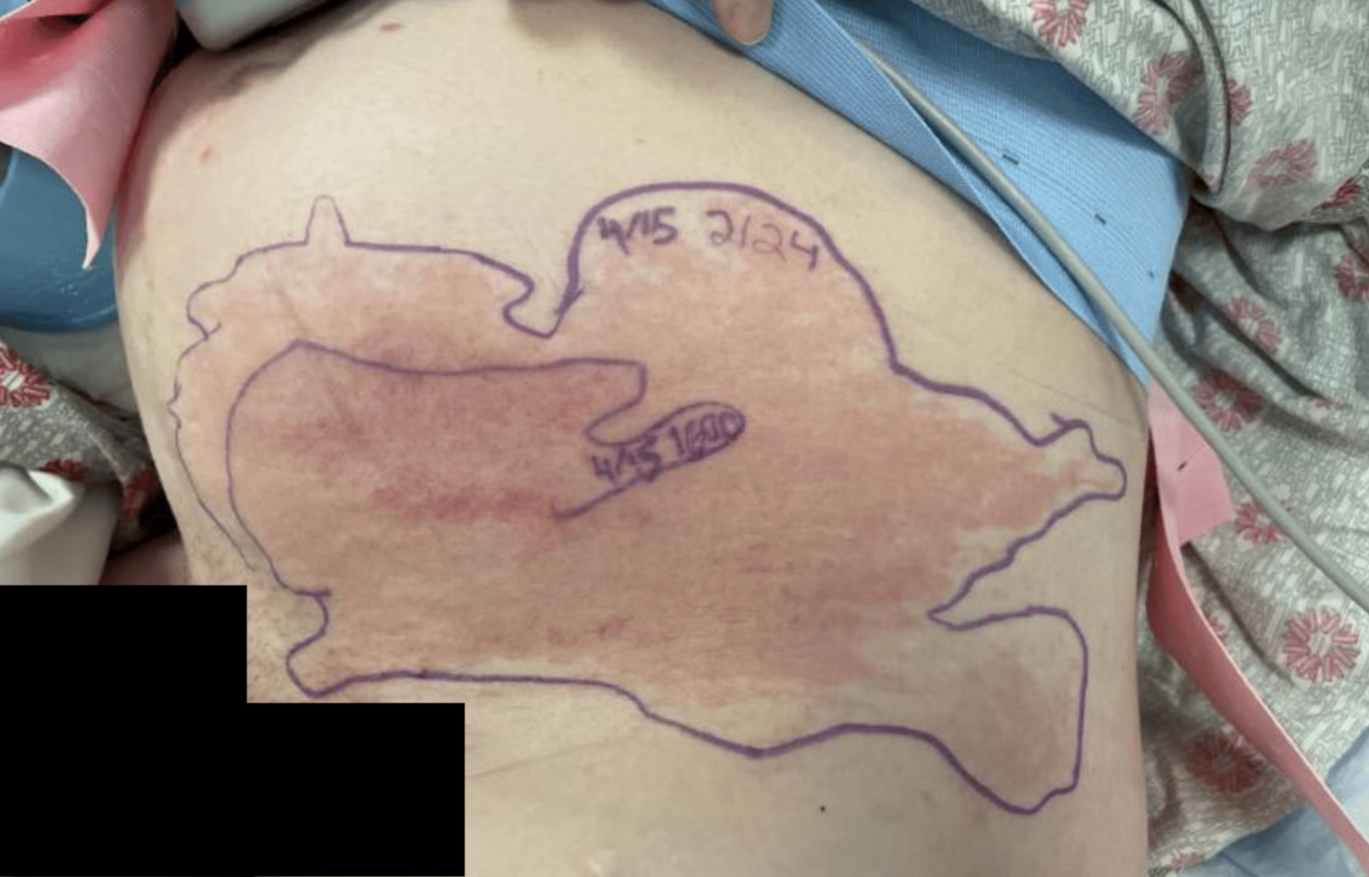
Photograph of the rash taken at the time of presentation

Presenting vitals were a temperature of 37.3 °C, a blood pressure of 95/58 mm Hg, a heart rate of 116 beats per minute, and a respiratory rate of 16 breaths per minute with an SpO₂ of 100% on room air. Physical examination revealed an erythematous, tender, ecchymotic-appearing rash in the left lower quadrant of the abdomen extending down the left thigh with absent crepitus (Figure 2). Left labial edema and erythema were noted in addition to a small pustule on the left side of the pubic symphysis. Lower extremities were positive for +1 pretibial pitting edema bilaterally. A cervical exam revealed 2 cm dilation, 50% effacement, and -3 station. Fetal heart tracing was category I, with tocometry showing contractions every two to three minutes. Laboratory studies were remarkable for leukocytosis of 18.7 k/mcL (Table 1) with neutrophil predominance of 88%, hemoglobin of 11.2 g/dL and hematocrit of 33.6%, platelet count of 231 k/mcL, mildly elevated alkaline phosphatase of 129 IU/L, erythrocyte sedimentation rate of 42 mm/hr, CRP of 7.4 mg/dL, procalcitonin of 0.09 ng/mL, and lactic acid of 0.9 mmol/L. A respiratory panel testing for COVID, influenza, and respiratory syncytial virus, in addition to a rapid group A streptococcus throat swab, yielded negative results. Urinalysis was inconsistent with a urinary tract infection. Two sets of blood cultures were collected. A rectovaginal GBS swab was performed due to concern for preterm labor.

**Table 1:** CBC throughout hospitalization Hct, hematocrit; Hgb, hemoglobin; MCH, mean corpuscular hemoglobin; MCHC, mean corpuscular hemoglobin concentration; MCV, mean corpuscular volume; RDW, red blood cell distribution width

Parameter	Reference range	Day 1	Day 2	Day 3	Day 4	Day 5
WBC	4.40-11.30 k/mcL	18.70 (↑)	24.82 (↑)	19.76 (↑)	18.27 (↑)	11
RBC	3.70-5.19 m/mcL	3.69 (↓)	3.15 (↓)	2.68 (↓)	2.68 (↓)	2.70 (↓)
Hgb	12.3-15.3 g/dL	11.2 (↓)	9.5 (↓)	8.1 (↓)	8.2 (↓)	10.5 (↓)
Hct	36.0-45.0%	33.6 (↓)	28.5 (↓)	25.0 (↓)	25.2 (↓)	25.5 (↓)
MCV	80.0-96.0 fL	91.1	90.5	93.3	94	94.4
MCH	27.5-33.2 pg	30.4	30.2	31	30.4	29.5
MCHC	31.0-35.9 g/dL	33.3	33.3	32.4	32.9	32.2
RDW	11.3-15.3%	12.2	12.4	12.7	12.8	12.7
Platelets	144-445 k/mcL	231	194	178	223	210

**Figure 3:**
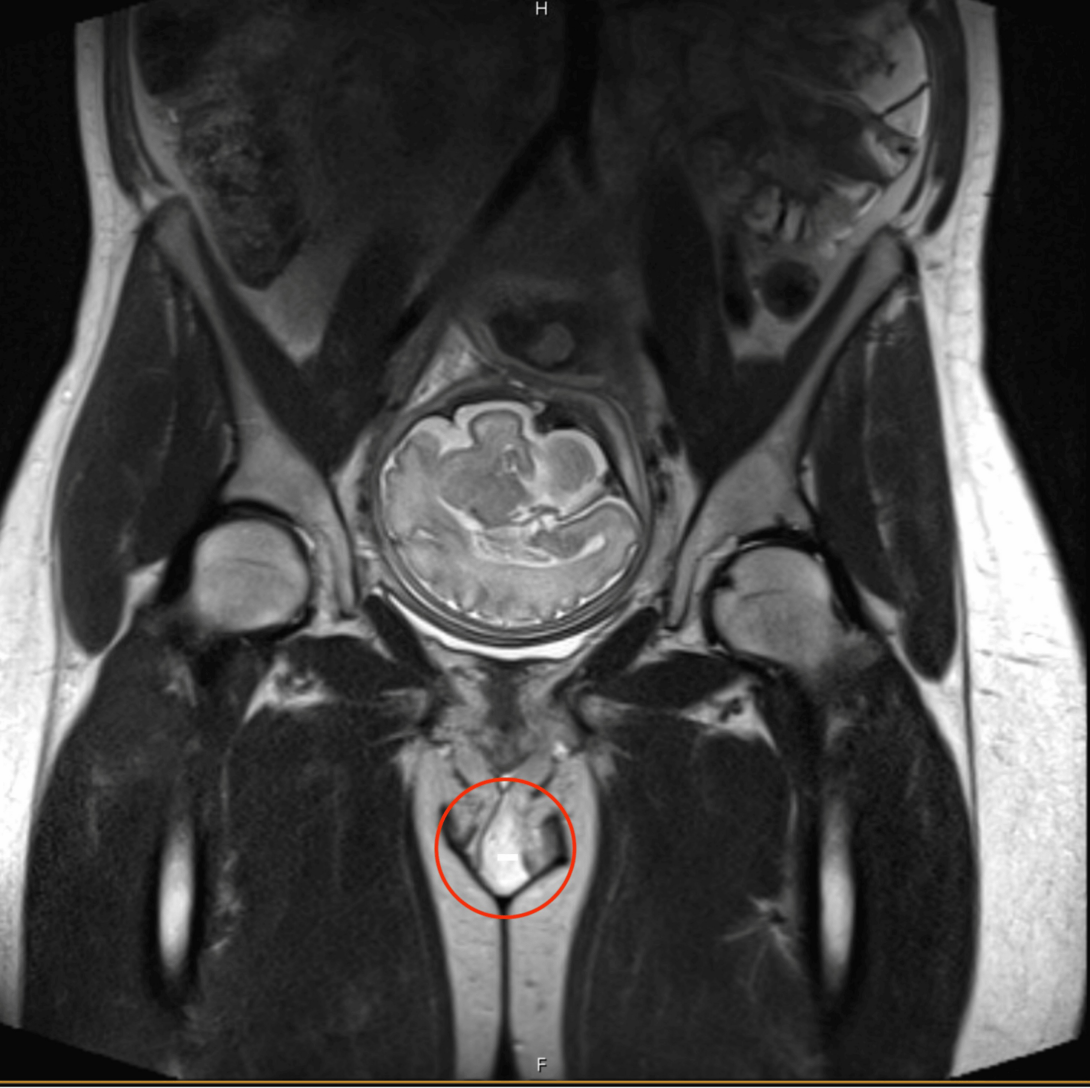
MRI of the pelvis without contrast Coronal view showing edematous labia. The image also demonstrates cephalic presentation of the fetus.

**Figure 4:**
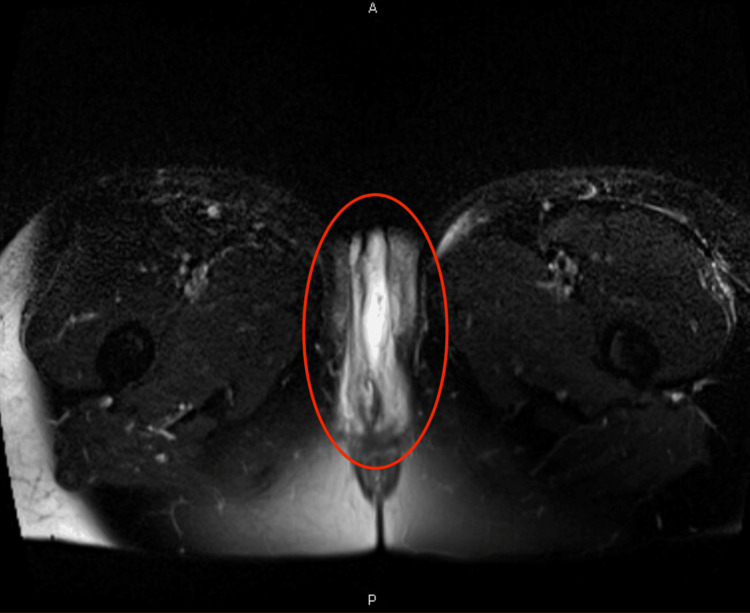
MRI of the pelvis without contrast Axial view showing bilateral labial edema.

**Figure 5:**
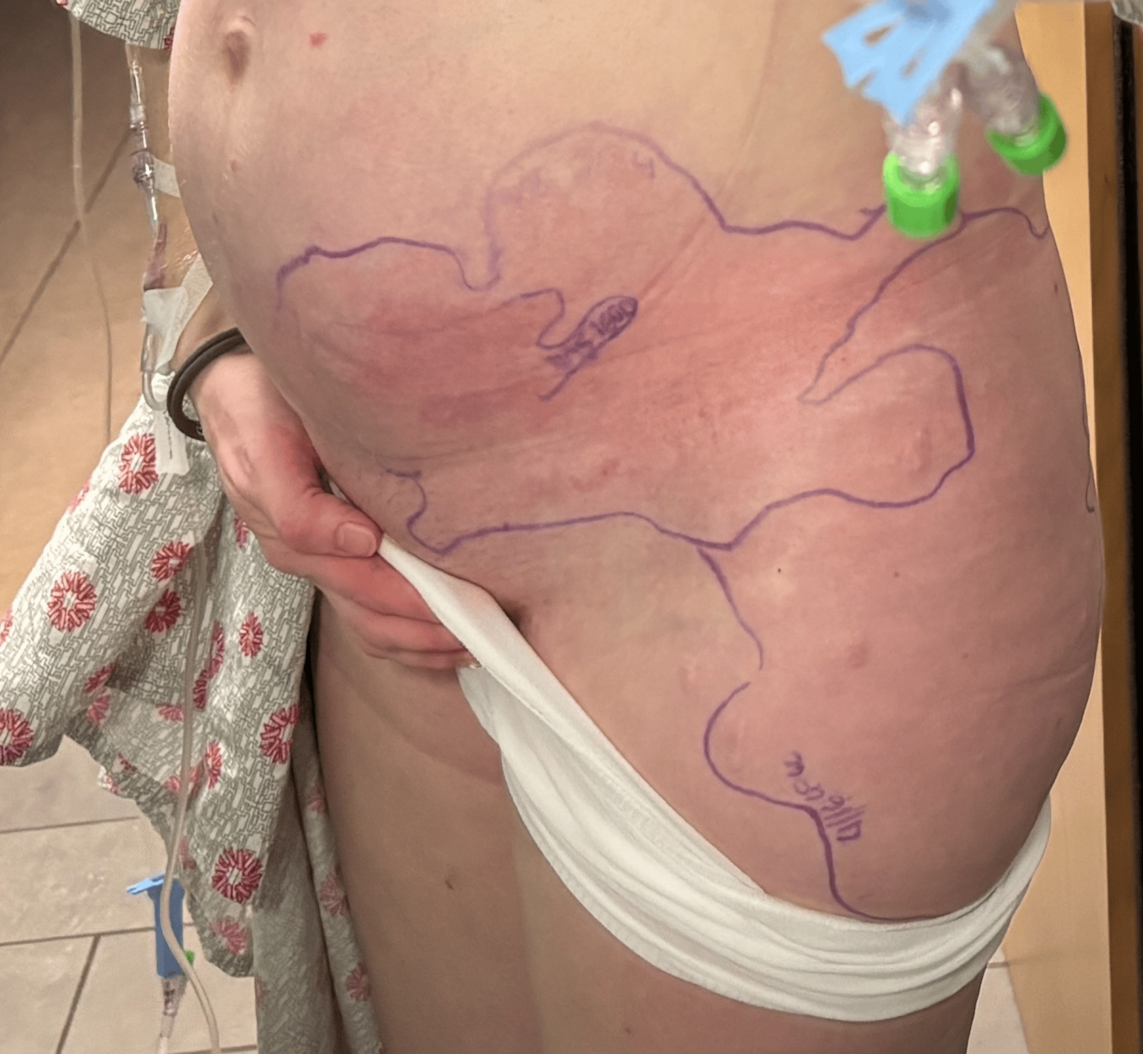
Photograph of the rash taken on hospital day 2

The patient’s initial tachycardia and leukocytosis raised concern for sepsis, prompting the initiation of empiric IV vancomycin with concomitant fluid resuscitation. Vancomycin was selected due to its efficacy against methicillin-resistant *Staphylococcus aureus*. The hospitalist service was consulted to further investigate the etiology of the rash, and diphenhydramine and topical hydrocortisone were added for symptomatic relief. Due to concerns for vulvar abscess, an MRI of the pelvis without contrast was ordered, which was significant for edematous labia and upper thighs without drainable fluid collection and a shotty right inguinal lymph node mildly increased compared to a prior scan in 2023, thought to be reactive (Figure 3, Figure 4). Dopplers of the lower extremity, ordered due to swelling and history of lymphedema, were negative for deep venous thromboses. During the first night of hospitalization, the patient developed a fever with a maximum temperature of 38.7 °C. The next morning, the rash was noted to have expanded to the left buttock and down the superolateral aspect of the left thigh (Figure 5). Preliminary blood cultures revealed Gram-positive cocci in chains. Infectious disease was consulted, and IV clindamycin and cefazolin were added to the current antibiotic regimen. Repeat blood cultures were also obtained. Cervical change was noted with a repeat cervical exam of 3 cm dilation, 90% effacement, and -1 station. Fetal heart tracing remained category I, and tocometry showed slowing contractions every three to five minutes. Intramuscular betamethasone was administered for the acceleration of fetal lung maturity, and neonatology was consulted in anticipation of preterm delivery.

On hospital day 3, initial blood cultures grew *S. agalactiae*, and a rectovaginal swab confirmed GBS colonization. Clindamycin and vancomycin were subsequently discontinued with the continuation of cefazolin. At this point, the patient’s rash began to subside. The cervical exam remained unchanged with category I fetal heart tracing. Contractions were no longer present. A second dose of intramuscular betamethasone was administered. A transthoracic echocardiogram, ordered to rule out endocarditis, demonstrated a normal ejection fraction of 60-64% with no gross density suspicious for thrombus, mass, or vegetation. On hospital day 5, repeat blood cultures remained negative. IV cefazolin was transitioned to cephalexin to complete a 14-day course, and the patient was later discharged with instructions to follow up with infectious disease in two weeks. At her follow-up appointment, the patient’s rash had resolved, and labial edema had returned to baseline. The patient proceeded to undergo an uncomplicated term vaginal delivery at 38 weeks and two days gestational age.

## Discussion

GBS is a well-known colonizer of the female rectovaginal tract and is extensively studied as a leading risk factor for neonatal sepsis. In the United States, the introduction of universal GBS screening in the 1990s has been associated with a significant decline in the rates of neonatal early-onset disease, including pneumonia, meningitis, and death [6]. This is mainly attributed to the high degree of accuracy in predicting maternal colonization at the time of delivery [6]. Current guidelines from ACOG recommend antepartum screening for GBS between 36 0/7 and 37 6/7 weeks of gestation, regardless of planned birthing modality [1]. Exceptions include a history of GBS bacteriuria during the current gestation or a history of a GBS infection in a newborn from a prior pregnancy, as IAP would be indicated in these cases regardless [1]. IAP is recommended for all women with positive cultures unless a prelabor cesarean section is performed prior to the rupture of membranes, with IV penicillin being the agent of choice and IV ampicillin as an acceptable alternative [7].

While current screening practices primarily exist to prevent neonatal disease, maternal infections, particularly invasive GBS infections, are less documented and may be under-recognized. Most maternal GBS infections during pregnancy are limited to urinary tract infections, chorioamnionitis, and endometritis [7]. However, more severe invasive clinical manifestations such as bacteremia, meningitis, and cellulitis have been documented in a small number of case reports and series [4]. Among neonates born to mothers with confirmed bacteremia, the incidence of early-onset neonatal sepsis reached 18.6%, underscoring the significant risk posed by invasive maternal infections [8]. In contrast, the incidence of early-onset GBS disease among the general US population is approximately 0.34-0.37 per 1,000 live births, reflecting the effectiveness of current prevention strategies in colonized non-bacteremic mothers [9].

While rare, invasive GBS disease poses a significant risk to the mother and fetus and is associated with a pregnancy loss rate of 22% [10]. Several systematic investigations have been conducted to determine the incidence of invasive GBS disease, which has been reported to range from 0.03% to 0.1% of all pregnancies [4,10]. A multistate evaluation found the incidence of invasive GBS disease to be twice as high in pregnant women compared to nonpregnant women, further emphasizing the heightened risk during pregnancy [11].

This case documents a unique presentation of invasive maternal GBS, demonstrated by culture-confirmed GBS bacteremia in the context of GBS cellulitis. At the time of presentation, the patient’s colonization status was unknown. The presence of cellulitis and bacteremia with associated systemic symptoms of fever, myalgia, and leukocytosis raised concern for sepsis. She was appropriately managed with empiric broad-spectrum IV antibiotics consisting of clindamycin, vancomycin, and cefazolin. Once blood cultures returned positive for GBS, her regimen was de-escalated to cefazolin monotherapy with eventual transition to oral cephalexin on discharge. The majority of cellulitis cases are initially treated empirically to cover for staphylococcal and streptococcal species. Once an infection is known to be caused by GBS, preferred treatment options include penicillin VK 500 mg oral every six hours, amoxicillin 500 mg oral every eight hours, or cephalexin 500 mg oral four times daily [12]. This patient was treated in accordance with these recommendations. Due to the fact that the patient’s pregnancy was complicated by GBS colonization, she did require IAP.

Of the invasive GBS infections, skin and soft tissue manifestations are the most commonly reported, including cellulitis, decubitus ulcers, and infected foot ulcers [5]. In particular, cellulitis has a predilection for individuals with predisposing vascular or lymphatic insufficiency, such as in this patient with a history of primary lymphedema and varicosities [5].

Demographic and clinical comparisons between GBS-negative, GBS-colonized, and invasive GBS cohorts revealed several significant associations. Compared to GBS-negative women, GBS-colonizers were more likely to be Black or African American (45.7% vs. 31.6%), have preexisting diabetes (3.5% vs. 2.8%), and have a history of tobacco use (20.8% vs. 17.8%), all statistically significant (p < 0.001 for all) [4]. Compared to colonized women, those with invasive disease were more likely to have chronic hypertension (25.0% vs. 8.6%) and preexisting diabetes (15.6% vs. 3.5%), all statistically significant (p < 0.001) [4]. These findings suggest that underlying metabolic and vascular comorbidities may predispose colonized individuals to progression toward invasive infection [4].

Recognizing the potential for invasive maternal disease, especially in patients with high-risk factors, highlights the importance of timely and appropriate management of rectovaginal colonizers. Although the primary intent of GBS screening remains that of neonatal prevention, it is important to remain cognizant of atypical maternal manifestations, as illustrated in this case. Invasive maternal GBS infection poses the mother and fetus at greater risk of complications and therefore poorer pregnancy outcomes. Expanding clinical awareness of potential GBS manifestations will help guide earlier recognition of invasive disease and allow treatment to be tailored to the patient’s specific condition, ultimately improving outcomes for both the mother and baby.

## Conclusions

This case of invasive GBS infection manifesting as bacteremia and concomitant cellulitis emphasizes the need for heightened awareness regarding atypical presentations. While the primary intention of GBS screening remains that of neonatal prevention, broadening our understanding of maternal GBS factors can improve early identification and appropriate management. Continued vigilance in the detection and treatment of GBS-related maternal infections is essential to reducing both maternal and neonatal complications. Ongoing documentation of rare manifestations is vital for increasing clinical awareness and subsequently improving outcomes.
